# Complete Genome Sequence of Lanthionine-Producing Lactobacillus brevis Strain 100D8, Generated by PacBio Sequencing

**DOI:** 10.1128/MRA.01220-18

**Published:** 2018-10-11

**Authors:** Min-Jung Kim, Hye Sun Kim, Sam Churl Kim, Youn-Sig Kwak

**Affiliations:** a Department of Plant Medicine, Institute of Agriculture and Life Science, Gyeongsang National University, Jinju, Republic of Korea; b Division of Applied Life Science (BK21Plus), Gyeongsang National University, Jinju, Republic of Korea; Queens College

## Abstract

Lactobacillus brevis strain 100D8 was isolated from rye silage and showed rapid acidification ability *in vitro* and antifungal activity against mycotoxin-producing fungi. We report here the complete genome sequence of L. brevis strain 100D8, which has a circular chromosome (2,351,988 bp, 2,304 coding sequences [CDSs]) and three plasmids (45,061 bp, 57 CDSs; 40,740 bp, 40 CDSs; and 39,943 bp, 57 CDSs).

## ANNOUNCEMENT

Silage fermentation consists of four phases, the initial aerobic phase, the fermentation phase, the stable storage phase, and the feed-out phase (1). During the aerobic phase, unwanted aerobic bacteria, yeast, and mycotoxins can accumulate in the silage (2). Lactic acid bacteria (LAB) have significant potential to overcome this difficulty because they have high dry matter digestibility as well as antifungal activity (3). Fungal growth inhibition by LAB is correlated with gene-encoded peptides (4). Moreover, lanthionines produced by LABs, such as nisin, lacticin 481, lactococcin, lactocin S, and carnocin, have been shown to inhibit the growth of mycotoxin-producing fungi such as Aspergillus, Penicillium, and Fusarium species (5). In order to identify LAB strains that might improve silage quality, Secale cereal silage samples were collected at 0, 1, 4, 7, 48, and 100 days during the fermentation period. Serially diluted samples in distilled water were spread on MRS medium and incubated at 27°C for 3 days. A total of 842 LAB colonies were isolated and stored as glycerol stocks for further experiments. An antifungal assay was performed to inhibit the mycotoxin-producing fungus, and rapid acidification ability was assessed for the basic process of silage fermentation. All the isolated colonies were incubated at 27°C for 3 days, and Fusarium moniliforme strain KACC 41032 was incubated as the mycotoxin-producing fungus for 7 days (6). The pH changes were determined from the mock-inoculated and inoculated bacteria in the MRS broth at intervals of 10 h (7). Among the isolates, Lactobacillus brevis 100D8 showed exceptional antifungal activity and rapid acidification ability. Therefore, to understand the genetic and biochemical characteristics of L. brevis strain 100D8, a complete genome sequence was obtained and analyzed in this study.

L. brevis strain 100D8 was incubated on MRS medium at 37°C for 2 days. Genomic DNA was extracted with a DNeasy UltraClean microbial kit (Qiagen). A complete genome sequence of L. brevis strain 100D8 was acquired with PacBio single-molecule real-time (SMRT) sequencing on the PacBio RS II platform at Macrogen Co. Ltd. (Seoul, Republic of Korea) (8). The sequencing library was prepared by random fragmentation. Sequencing data were converted into raw data for the analysis, and a quality check of raw sequences was performed using FastQC (http://www.bioinformatics.babraham.ac.uk/projects/fastqc). *De novo* assembly was conducted using SOAPdenovo2 version 2.04 with the Fast Alignment and CONsensus (FALCON) version 0.2.1 tool. The filtered data generated a total of 2,177,050,960 bases and 21,554,960 reads in five contigs. After the whole genome was assembled, gene functions were annotated using Prokka version 1.11 (9). The genome of L. brevis strain 100D8 comprises a single chromosome (2,351,988 bp) and three plasmids (45,061 bp, 40,740 bp and 39,943 bp). The G+C contents of these components are 46.1%, 38.4%, 38.7%, and 40.8%, respectively. This strain carries 65 tRNAs and 15 rRNAs. The chromosome sizes of L. brevis strain 100D8 and L. brevis ATCC 367 are similar, but the three plasmids in strain 100D8 are larger than those in strain ATCC 367 (9). The genomic information was analyzed to predict putative antibiotic gene clusters and biosynthetic genes through antiSMASH version 3.0.5, Rapid Annotations using Subsystems Technology (RAST) version 2.0, and NCBI annotation systems. Of 2,499 predicted coding sequences, 1,593 were classified into different functional categories based on the subsystem category distribution. Most of the genes in L. brevis strain 100D8 were associated with carbohydrate and protein metabolism, cell wall biosynthesis, and capsulation. The antiSMASH pipeline predicted eight putative antibiotic biosynthesis genes. In particular, the *lanC* gene encoding a lanthionine synthetase C-like protein was present in cluster 1. LanC has known antifungal activity against mycotoxin-producing fungi (10). The *lanC* gene in strain 100D8 was expressed at 2.7 to 3.8 log copy numbers/μg total RNA relative to the *pheS* and *rpoA* housekeeping genes (11).

### Data availability.

L. brevis strain 100D8 has been deposited in the Korean Culture Center of Microorganisms under the accession number KCCM11787P. The complete genome sequences for the silage were deposited in GenBank under BioProject number PRJNA318943, BioSample number SAMN04870420, SRA number SRR7820376, and accession numbers CP015338 for the chromosome, CP015339 for plasmid 1, CP015340 for plasmid 2, and CP015341 for plasmid 3.
